# Impact of Hypoglycemia on Hospitalized Patients With Hepatocellular Carcinoma

**DOI:** 10.7759/cureus.64673

**Published:** 2024-07-16

**Authors:** Rabia Iqbal, Divya Solipuram, Yaqub Nadeem Mohammed, Ahmad Taimoor Bajwa, Arslan Irfan, Amina Jafar, Zarlish Rehman, Zaigham Ul Islam

**Affiliations:** 1 Medicine, The Brooklyn Hospital Center, Brooklyn, USA; 2 Internal Medicine, Nassau University Medical Center, East Meadow, USA; 3 Internal Medicine, Western Michigan University Homer Stryker M.D. School of Medicine, Kalamazoo, USA; 4 Oncology, Brooklyn Cancer Care, Brooklyn, USA; 5 Internal Medicine, University of Pittsburgh Medical Center, Pittsburgh, USA; 6 Oncology, Shaukat Khanum Memorial Cancer Hospital and Research Centre, Lahore, PAK; 7 Internal Medicine, Hayatabad Medical Complex, Peshawar, PAK; 8 Internal Medicine, Nishtar Medical University, Multan, PAK

**Keywords:** hepatocellular carcinoma, malignant ascites, hepatic failure, life prognosis, hepatocellular carcinoma trends outcomes in-hospital mortality, hypoglycemia

## Abstract

Aims

Hepatocellular carcinoma (HCC) is one of the common liver malignancies that presents a challenge to global healthcare. The impact and outcomes of hypoglycemia in HCC have not been studied in detail before. This study aimed to investigate the outcomes and prognosis associated with hypoglycemia in patients diagnosed with HCC, utilizing a large-scale database approach.

Methods

Using the Nationwide Inpatient Sample (NIS) database from 2017 to 2020, we conducted a comprehensive retrospective analysis to examine the incidence, risk factors, and clinical implications of hypoglycemia on HCC patients. The patients were divided into two groups: those with hypoglycemia and those without hypoglycemia. Univariate and multivariate logistic regression were used to conduct the analysis. STATA® version 17.0 software (StataCorp LLC, College Station, TX) was used for this purpose.

Results

Out of a total of 343,895 patients with HCC, the prevalence of hypoglycemia was present in 1.5% of this patient population. We found that hypoglycemia was common in the male population (68%). Compared with patients without hypoglycemia, patients who had hypoglycemia with HCC had higher mortality (42%, p-value < 0.05) and higher risks of secondary outcomes such as hepatic failure, spontaneous bacterial peritonitis (SBP), ascites, and portal vein thrombosis compared to patients who did not have hypoglycemia. The multivariate-adjusted odds ratio for hepatic failure was 2.7 (2.3-3.1), for SBP was 2.9 (1.8-3.0), for ascites was 1.6 (1.4-1.9), and for portal vein thrombosis was 1.2 (0.9-1.4).

Conclusion

In conclusion, hypoglycemia in HCC is associated with increased mortality and worse outcomes.

## Introduction

Hepatocellular carcinoma (HCC) poses a significant global health challenge, ranking prominently among primary liver malignancies worldwide [1,2]. The incidence of hepatocellular cancer has more than tripled since 1980, with a corresponding doubling of mortality rates during the same period, according to the American Cancer Society [3]. Despite notable advancements in treatment, effectively managing HCC remains a formidable task due to its aggressive nature and intricate interaction with various bodily processes, including glucose metabolism [4].

The presence of hypoglycemia is associated with lesser survival in HCC and cirrhosis patients [5]. Since it was first reported by Nadcer et al. in 1929, hypoglycemia associated with HCC has gained more attention [6]. It is a common complication in HCC, which can occur due to two different mechanisms. The first occurs due to hepatocyte damage, disrupting glycogenolysis and gluconeogenesis. Hence, the presence of hypoglycemia acts as a prognostic indicator of mortality in HCC patients [7]. The second one is a paraneoplastic syndrome, also known as non-islet cell tumor hypoglycemia (NICTH) [8]. HCC has been associated with NICTH and can lead to severe complications. Hypoglycemia in HCC can be of two types: type A, which occurs in the last stage of the disease, characterized by rapid growth of the tumor. Mortality occurs within two weeks. Type B hypoglycemia, on the other hand, is associated with slow-growing tumors [9,10], and hypoglycemia typically appears two to 10 months before death.

Despite its clinical relevance, the precise implications of hypoglycemia on the prognosis and clinical trajectory of HCC patients remain uncertain. In this study, we aimed to investigate the outcomes and prognosis associated with hypoglycemia in patients diagnosed with HCC, utilizing a large-scale database approach. Through our research, we strive to fill a significant gap in the current understanding of the prevalence of hypoglycemia and its impact on outcomes in HCC patients across diverse demographic groups.

## Materials and methods

Study data

The data were obtained from the Nationwide Inpatient Sample (NIS) dataset from 2017 to 2020. NIS is the most extensive inpatient dataset in the United States, and it is sponsored by the Agency for Healthcare Research and Quality. The aim of establishing the NIS was to compile databases to analyze national trends in healthcare utilization, healthcare quality, and patient outcomes. Approximately 35 million discharges are evaluated annually, offering an accurate national representation while maintaining a substantial sample size. It can provide us with a wide range of information, including patient demographics, comorbidities and severity of illness, hospital characteristics, insurance information, and outcomes of different diseases. The Healthcare Cost and Utilization Project (HCUP) comprises data from hospitals and institutions worldwide. Researchers then access the dataset through institution agreements and standardized protocols to ensure data extraction and analysis methodologies are consistent across geographical regions. Through this collaboration, authors from different institutions can comprehensively analyze healthcare trends across diverse settings nationwide. It contains confidential data regarding patients and hospitals, while de-identified information regarding clinical practices and resource utilization is made available. As the information is de-identified, IRB approval was waived.

Study design and population

In this study, we selected patients aged > 18 years with a primary diagnosis of HCC and a secondary diagnosis of hypoglycemia using the NIS database from 2017 to 2020. Patient detection was based on the International Classification of Diseases, Tenth Revision, Clinical Modification (ICD-10-CM) codes. Patients with HCC admitted to the hospital were diagnosed with hypoglycemia during the same hospital visit. Hospitalizations were divided into two groups: HCC with hypoglycemia and without hypoglycemia.

Study variables and outcomes

The primary outcomes included mortality, length of stay, and cost of hospitalization. The secondary outcomes included altered mental status, arrhythmias, seizures, portal vein thrombosis, ascites, variceal bleeding, hepatic failure, spontaneous bacterial peritonitis, obstructive jaundice, and portal hypertension. We used baseline characteristics, including patient demographics, including age (in years), gender, race, BMI, median household income, hospital teaching status (rural, urban non-teaching, and urban teaching hospitals), and hospital bed size (small, medium, and large). Confounding variables were adjusted using multivariate regression analysis, which included the use of alcohol, recreational drugs, history of smoking, diabetes, hypertension, dyslipidemia, chronic kidney disease, chronic pulmonary disease, coronary artery disease, and congestive heart failure.

Statistical analysis

We used STATA® version 17.0 (StataCorp LLC, College Station, TX) software for data analysis. Continuous data were presented as mean, while categorical data were presented as percentages and frequencies. Student's t-test was used to analyze continuous variables, while the chi-square test was used for categorical variables. Univariate logistic regression was used to calculate the unadjusted odds ratio (OR) for primary and secondary outcomes. Multivariate logistic regression was used at the end of the final analysis to adjust for confounding factors. The included confounders included demographic characteristics, smoking history, alcohol use, opioid use, hypertension, dyslipidemia, diabetes, chronic kidney disease, chronic pulmonary disease, congestive heart failure, and coronary artery disease. P-values were two-sided and statistical significance was set at P-value < 0.05.

## Results

From 2017 to 2020, 343,895 patients were hospitalized in the United States with a primary diagnosis of HCC. Approximately 33% (n = 113,485) of these patients had cirrhosis. Within the subset of patients with cirrhosis, a smaller percentage of 0.9% (n = 1022) experienced hypoglycemia. On the other hand, 1.5% (n = 5159) experienced hypoglycemia without cirrhosis. The top three reasons for hospital admission included metastatic spread to the biliary duct and related symptoms, sepsis, and liver failure (Table 1).

**Table 1:** Reason for hospital admission by proportion.

Cause of hospital admission	No. of patients	Proportion
Metastasis to biliary system	30950	9%
Sepsis	27512	8%
Hepatic failure	13756	4%

Men were more affected (68%) than women (32%), though the gender difference was not statistically significant (p = 0.06). The mean age of hypoglycemic patients was 64 years. The White race had the highest prevalence of hypoglycemia (46%), while Native Americans had the lowest (2%). Hypoglycemia was more common in the lowest household income quartile (35%) and more prevalent in urban teaching hospitals (p = 0.01). Furthermore, hypoglycemia was prevalent in HCC patients with a BMI ranging from 20 to 40 (15%, p-value < 0.001), followed by those with a BMI of 19 or less (10%, p-value < 0.01), and least common in patients with a BMI over 40 (1.7%, p-value = 0.02) (Table 2).

**Table 2:** Patient demographics, household income, hospital bed size, and teaching status comparing the difference between hepatocellular carcinoma with and without hypoglycemia. Student's t-test was used to calculate age, while the chi-square test was used to calculate the remaining variables.

Patient characteristics	With hypoglycemia	Without hypoglycemia	P-value
Age (mean) (years)	64	66	<0.001
Gender			
Female (%)	32	33	0.6
Male (%)	68	67	0.6
Race			
White	46	60	<0.001
Black	25	13	<0.001
Hispanic	14	16	<0.001
Asian	9	6	<0.001
Native American	2	1	<0.001
Other	4	4	<0.001
Median household income (percentile)			
0-25th	35	30	0.02
26th to 50th	24	25	0.02
51st to 75th	22	24	0.02
76th to 100th	19	21	0.02
Hospital status			
Rural	3	5	0.02
Urban, non-teaching	13	15	0.02
Urban, teaching	84	80	0.02
Hospital size			
Small	17	16	0.01
Medium	29	25	0.01
Large	54	59	0.01

Patients with hypoglycemia had higher all-cause mortality (adjusted odds ratio (aOR): 6.9, p < 0.001). Additionally, these patients were more likely to have conditions such as hepatic failure (aOR: 2.7, p < 0.001), spontaneous bacterial peritonitis (aOR: 2.9, p < 0.001), ascites (aOR: 1.6, p < 0.001), and portal vein thrombosis (aOR: 1.2, p < 0.001). Although higher odds of altered mental status, seizure, and variceal bleeding were observed, these were not statistically significant (p > 0.05). Although the association of altered mental status (AMS) with hypoglycemia was not significant, the analysis revealed that AMS associated with cirrhosis (hepatic encephalopathy) was significant (p = 0.02) (Table 3).​​

**Table 3:** Outcomes comparing the difference in hepatocellular carcinoma patients with and without hypoglycemia. Student's t-test was used to calculate the p-values.

Frequency (%)	With hypoglycemia	Without hypoglycemia	Adjusted odds ratio and confidence interval	P-value
Mortality	42	8.7	6.9 (6.0-8.0)	<0.001
Hepatic failure	42	21	2.7 (2.3-3.1)	<0.001
Spontaneous bacterial peritonitis	8	3	2.9 (1.8-3.0)	<0.001
Ascites	35	23	1.6 (1.4-1.9)	<0.001
Portal vein thrombosis	13	10	1.2 (0.9-1.4)	0.04
Variceal bleeding	4	3	1.1 (0.8-1.5)	0.4
Altered mental status	1.1	0.8	0.3 (0.7-2.4)	0.3
Arrhythmias	8.3	9.6	1.0 (0.8-1.3)	0.6
Seizures	0.3	0.1	2.6 (0.-8.8)	0.1
Obstructive jaundice	8.5	9.2	1.0 (0.8-1.3)	0.7

## Discussion

Out of the 343,895 patients admitted with HCC between 2017 and 2020 in the United States, 1.5% of patients had hypoglycemia without cirrhosis. Almost 33% of HCC patients had cirrhosis, and 0.9% had hypoglycemia. A retrospective study of 534 HCC patients done between 2010 and 2020 reported a 22% incidence of paraneoplastic syndrome (PNS), out of which 5.8% of patients had hypoglycemia [11]. Our study found that patients with hypoglycemia were younger (64 vs. 66) as compared to HCC patients without hypoglycemia. A mean age of 47.0 ± 8.0 years was reported in a systematic review of 21 patients of HCC with hypoglycemia [12]. The mean age reported in this review was lower than ours, but it only included a limited sample size.

Our study found that males had a higher prevalence of HCC than females, a trend that was observed in both patients with and without hypoglycemia. This finding is consistent with a review of 11 case reports on hypoglycemia in HCC between 1956 and 2011, which showed that 82% of the cases were male, similar to our study's population composition of 68% males [13]. We also found that the prevalence of hypoglycemia was higher among White patients compared to other racial groups. This is consistent with a previous study using the same NIS database, which reported a 54% White population and 16% Hispanic and Black population for HCC between 2008 and 2017 [14].

As mentioned in the introduction, hypoglycemia in HCC is classified into two types: type A, characterized by rapidly progressive tumors with significant muscle weakness and wasting; and type B, associated with slow-growing tumors and minimal muscle weakness and wasting. In our study using the NIS database, it was not feasible to differentiate between these types of hypoglycemia due to the lack of detailed clinical data. While the database can diagnose hypoglycemia, it does not provide specific information on the type. The pathogenesis of hypoglycemia in HCC is not well understood, as it is much less common in solid tumors. Potential mechanisms include decreased gluconeogenesis due to the destruction of liver parenchyma by large tumors or excess insulin production from non-islet cell tumors such as insulinomas. Additionally, HCC cells may have high metabolic demands, consuming large amounts of glucose and resulting in hypoglycemia [15].

In our analysis, hypoglycemia in HCC was also found to be associated with higher all-cause inpatient mortality. This finding aligns with previous literature indicating that hypoglycemia is linked to unfavorable outcomes and typically occurs at advanced stages of the disease [15]. Additionally, our study showed that hypoglycemia in HCC is associated with an increased risk of other outcomes, including hepatic failure, SBP, ascites, and portal vein thrombosis. HCC can present with hepatic failure symptoms such as jaundice, fever, abdominal pain, pruritus, weight loss, and lethargy, and can also directly cause liver failure, particularly post-hepatectomy due to loss of compensatory functions [16]. Hepatic failure leads to features of decompensated liver disease, like ascites due to low albumin production, defective coagulation due to the inability of the liver to make coagulation proteins, and portal vein thrombosis caused by direct invasion of the vessels by tumor [17]. In our study, 35% of HCC patients with hypoglycemia had ascites (p = 0.00), compared to 23% without hypoglycemia. A study done in Taiwan that collected data over eight years, including 2203 patients with HCC, found that 498 (23%) of the patients had developed ascites [18]. Liver dysfunction in HCC patients also compromises immunity, increasing the risk of recurrent infections like SBP [19]. A similar finding was found in a prospective study performed over two and a half years, which included 262 patients, which showed that the in-hospital incidence and mortality from SBP in HCC patients were 7.3% and 50% (p < 0.001), respectively [20].

Clinical implications/future direction

Over the past few decades, the evidence on hypoglycemia in HCC has been limited to case reports and case series only, which cannot be extrapolated to the general population for formulating guidelines and protocols. Our study contributes to the existing literature by investigating the prevalence, demographic features, and outcomes of hypoglycemia in HCC patients using a large, nationally representative sample.

Strengths

The large sample size of our study provides a broad representation of patients in US hospitals, enhancing the external validity of the results. To our knowledge, this is the first national-level study to explore the association between hypoglycemia and HCC. Additionally, our comprehensive analysis offers valuable insights into various socioeconomic and baseline demographic characteristics, which are often challenging to assess in single-institution studies. Furthermore, this study highlights differences in the prevalence of hypoglycemia among different racial groups, shedding light on healthcare disparities and emphasizing the need for targeted interventions.

Limitations

In this study, we utilized the NIS database, which introduces limitations, including coding errors, missing data, and inaccuracies. Although the study does have a large sample size to allow for some degree of generalization of the results, hypoglycemia remains an uncommon complication of HCC (only 1.5% of patients in our study). This might be due to underreporting in databases. Our study relies solely on inpatient data, making it difficult to generalize the findings to the outpatient setting. As the NIS database contains administrative data, it lacks the detailed clinical information and follow-up necessary to establish cause-and-effect relationships between variables. Lastly, the results could be influenced by the severity of acute illness, which is more prevalent in hospitalized patients. By explicitly acknowledging these limitations, we aim to provide a more transparent and accurate interpretation of our findings.

## Conclusions

Hypoglycemia, a recognized complication of HCC, is explored in our retrospective study. Through comprehensive analysis of the NIS database, we found that hypoglycemia in HCC is associated with higher mortality and worse secondary outcomes such as hepatic failure, ascites, spontaneous bacterial peritonitis, and portal vein thrombosis. Further research and clinical efforts are needed to understand the underlying mechanisms, aiding in risk stratification, early identification, and the development of appropriate management strategies to improve survival and patient outcomes.
